# Spatial Vulnerability: Bacterial Arrangements, Microcolonies, and Biofilms as Responses to Low Rather than High Phage Densities

**DOI:** 10.3390/v4050663

**Published:** 2012-04-26

**Authors:** Stephen T. Abedon

**Affiliations:** Department of Microbiology, The Ohio State University, 1680 University Dr., Mansfield, OH 44906, USA; Email: abedon.1@osu.edu; Tel.: +1-419-755-4343; Fax: +1-419-755-4327

**Keywords:** adsorption, bacteriophage, biofilms, cellular arrangements, ecology, microcolonies, particle transport, phages, phage therapy

## Abstract

The ability of bacteria to survive and propagate can be dramatically reduced upon exposure to lytic bacteriophages. Study of this impact, from a bacterium’s perspective, tends to focus on phage-bacterial interactions that are governed by mass action, such as can be observed within continuous flow or similarly planktonic ecosystems. Alternatively, bacterial molecular properties can be examined, such as specific phage‑resistance adaptations. In this study I address instead how limitations on bacterial movement, resulting in the formation of cellular arrangements, microcolonies, or biofilms, could *increase* the vulnerability of bacteria to phages. Principally: (1) Physically associated clonal groupings of bacteria can represent larger targets for phage adsorption than individual bacteria; and (2), due to a combination of proximity and similar phage susceptibility, individual bacteria should be especially vulnerable to phages infecting within the same clonal, bacterial grouping. Consistent with particle transport theory—the physics of movement within fluids—these considerations are suggestive that formation into arrangements, microcolonies, or biofilms could be either less profitable to bacteria when phage predation pressure is high or require more effective phage-resistance mechanisms than seen among bacteria not living within clonal clusters. I consider these ideas of bacterial ‘spatial vulnerability’ in part within a phage therapy context.

## 1. Introduction

Environments can be distinguished in terms of the degree of spatial structure that they exhibit, where spatial structure is a description of the extent to which diffusion, motility, and environmental mixing are constrained. Important spatially structured bacterial habitats include soils; sediments; surface tissues of plants, animals, and fungi; and bacterial biofilms in general. The latter are found both as suspended aggregates and on most submerged surfaces. It is an oft-repeated assertion that the majority of bacteria, or at least a large fraction, may be found within biofilms rather than as planktonic organisms [1,2]. Naturally occurring bacteria thus exist to a great extent as spatially structured populations or communities. Furthermore, and pertinent to fields as diverse as medicine [3] and civil engineering [4], pathogenic or nuisance bacteria found in the biofilm state can be resistant to both antibiotics and disinfectants—a resistance that can be more a function of the phenotypic plasticity of bacteria, *i.e.*, varying metabolic states, rather than due to either genetically acquired resistance or diffusion barriers to chemical penetration into biofilms. Development of alternative methods of biofilm removal therefore is desirable [5].

Bacteriophages, the viruses of bacteria, are a possible alternative to antibiotics, or disinfectants, as antibacterial agents. Such phage therapy or phage-mediated bacterial biocontrol [6,7] has shown promise against bacterial biofilms [8,9]. Rather than a relatively new aspect of phage study, however, the exploration of phage infection of spatially structured bacterial populations goes back to the beginning of the phage era. The first generally recognized bacteriophage study [10], that of Twort [11,12], considered in particular the phage impact on bacteria growing as colonies. Though subsequent studies of phage interaction with macroscopic bacterial colonies have been relatively rare, observation of phage-induced lysis of microscopic colonies has been a routine facet of phage biology, with a growing literature considering explicitly the dynamics of bacterial lysis within the context of phage plaque formation [9,13,14,15,16,17]. Given the ubiquity of biofilms within natural environments, phage interaction with spatially structured bacterial populations should be somewhat relevant to our appreciation of phage environmental microbiology in general [13,18]. Similarly, improved understanding of such interactions may possess applied significance, such as helping to inform phage choice as anti-biofilm agents [19,20] or phage modification to improve anti-biofilm properties [21,22].

Here I explore the costs to bacteria of ‘group living’ that can result from exposure to phages. This I term a ‘spatial vulnerability’ because bacteria that are physically attached together—as arrangements, microcolonies, “macrocolonies” [23], or otherwise within biofilms—display less mobility relative to each other than do equivalent bacteria found as physically isolated cells. The result, if physically associated bacteria are clonally related [1], can be a greater negative impact resulting from phage exposure than if the same bacteria instead existed as free cells. I argue that benefits associated with group living therefore are accessible to bacteria only to the extent that their vulnerability to phages nevertheless is small. Murray and Jackson [24] provide comparable though more general arguments based on particle transport theory, that is, the physics of un-self-propelled movement within fluids as applied to aquatic viruses. In general, being large is possible only if the pressure of viral predation is sufficiently low.

Mechanisms that can reduce bacterial exposure to phages include existence within environments into which phage penetration is difficult or when bacteria exist at sufficiently low densities that they are unable to support phage amplification to “inundative” densities [25]. The latter can be described as an avoidance of ‘kill the winner’ mechanisms [26,27] or, equivalently, bacterial existence within numerical refuges [28,29]. A general implication is that biofilms may tend to persist particularly within environments in which the densities of phages targeting those bacteria are relatively low or, alternatively, that biofilm-forming bacteria *must* possess substantial phage-resistance mechanisms [30,31,32] in order to maintain their populations within environments where phage predation pressures are relatively high.

The common theme is that living as physically associated and therefore spatially structured clonal groups, in and of itself, should not be expected to serve bacteria as a phage-resistance mechanism. Rather, I argue from first principles that group living can result in greater bacterial vulnerability to phages than may be experienced by bacteria that instead are physically separated from their clonal relations. With this perspective in mind, the utility of phages as an anti-bacterial as well as a specifically anti-biofilm strategy may be appreciated as an explicit ecological reversal of exactly those circumstances in which biofilms otherwise may flourish: Application of sufficient densities of phages, where phages otherwise are lacking, such that uncontrolled bacterial proliferation can be reversed.

## 2. Results and Discussion

In this study the primary question being asked is what might be the ecological costs to bacteria, in light of phage predation, that are associated with bacterial growth as arrangements or microcolonies. To answer this question, I generally employ ecological models, arguments, and scenarios—that is, considerations of how bacteria may interact with their environments—and this is rather than primarily enlisting evolutionary approaches or perspectives. In addition, as the study represents a relatively novel exploration the ideas presented, I limit discussion to less complex scenarios, avoiding addressing for instance consideration of stochasticity, the growth in size of bacterial arrangements, or simulations of phage-bacterial ecological dynamics.

### 2.1. Phage Adsorption to Free Bacteria

The interaction between phages and those bacteria that exist as individual, planktonic cells—here, collectively, “free” bacteria—is fairly straightforward. Beginning with phage attachment to a susceptible bacterium, phage-genome uptake occurs, initiating the infection proper. At some point mature virions must be released from the infected bacterium, beginning an extracellular search for new bacteria to infect [33]. This search is driven by a combination of phage diffusion, fluid flow including environmental mixing, and bacterial as well as bulk environmental movement [18]. Upon sufficient mixing, all bacteria within an environment are then equally likely to encounter a particular phage that has just been released from a specific bacterium. That is, spatial structure can be said to largely *not* exist given a combination of bacteria that are “free” and substantial environmental mixing. In this section I consider the basics of phage adsorption, focusing particularly on issues of encounter rates between phages and bacteria rather than mechanisms of phage attachment or subsequent phage initiation of infection. For visualization of the spatial scale of environments in which these interactions take place, see Abedon [34].

#### 2.1.1. Phage Movement towards Bacterial Targets

**Figure 1:**
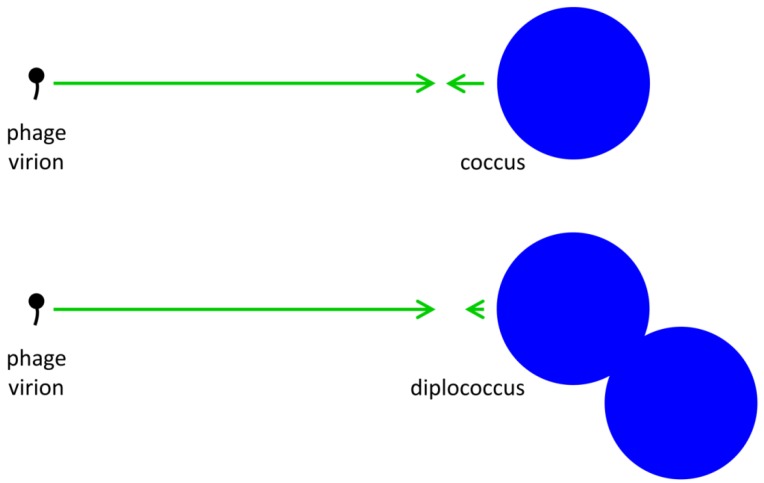
Illustration of phage and bacterial contributions to phage adsorption rates. Generally phages are relatively small and bacteria somewhat larger. Since diffusion rates are inversely proportional to particle size, whereas target size is proportional to particle size, the result is that phage diffusion (larger arrows pointing right) is a more important contributor to phage adsorption than is bacterial diffusion (smaller arrows point left) while bacterial target size is more important than phage target size to the likelihood of phage‑bacterial encounter. An approximate doubling of total bacterial size (lower right) consequently affects target size but has little relevant impact on combined diffusion rates. Note that arrow lengths reflect an assumption that phages are one-tenth the diameter of the coccus and one-twentieth the diameter of the diplococcus.

The extracellular search at its most basic consists of a process of virion diffusion. Such diffusion, due to the comparatively small size of phages, occurs at a rate that is substantially greater than the diffusion of free-floating bacteria. As a result, phage extracellular movement towards an idealized bacterium can be described [24] (p. 104) as “simple diffusion to a single sphere”. The likelihood of phage-bacterium collision, even in an environment lacking in spatial structure, thus is a function of phage diffusion much more so than the diffusion of target bacteria. Given the substantially larger size of bacteria relative to phages, the likelihood of phage-bacterium encounter is governed by bacterial target size much more so than phage diameter [35]. Free phages thus can be considered to rapidly diffuse among relatively large and stationary bacterial targets (Figure 1).

Rates of virus diffusion along with the size of target bacteria are not the only factors affecting rates of encounter between these entities, as so too do their environmental densities. For instance, and as considered in some detail by Murray and Jackson [24], the specific rate of virus adsorption is a function of both the size as well as number of adsorbable targets. Bacteria thus represent smaller targets relative to protozoa and as a consequence individual viruses are less likely to encounter individual bacteria in comparison to individual protozoa. Protozoa, however, tend to display lower population densities than do bacteria, resulting in lower rates of virus encounter with protozoa despite the latter’s larger individual sizes. These lower rates of encounter can have the effect of keeping prey densities below ‘winner’ concentrations (Section 1) despite larger target sizes for individual prey organisms.

Ioannou *et al.* [36] provide both theory and experimental evidence that, in light of predation, the costs to prey of increasing in size—and therefore becoming more absolutely visible to predators—may be offset by prey decrease in abundance. In particular, larval prey that are present at lower densities will be relatively less visible due to, on average, their existing at greater distances from their three‑spined stickleback predator (a type of fish), just as bacteria existing at lower densities too, on average, are present at greater distances from individual phages relative to bacteria found in higher density bacterial populations. Consistent with these considerations, in subsequent sections I suggest that bacterial existence as arrangements or as microcolonies can result in greater bacterial vulnerability to phages due to resulting increases in overall target size. I suggest in addition that such vulnerabilities may be avoided at least in part when bacterial populations are relatively rare, that is, should bacteria enter into what have been described, for free bacteria, as numerical refuges [28,29].

#### 2.1.2. Basic Adsorption Calculations

The higher the density at which phage virions are present within environments then the more likely that a given bacterium will encounter a phage, potentially resulting in bacterial conversion from phage uninfected to phage infected. Thus, 


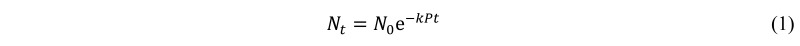


where *N_t_* is the density of bacteria that are not phage infected at the end of some interval (*t*), *N*_0_ the density of uninfected bacteria at the beginning of that interval, *P* is the density of free phages (that is, phages that are both unadsorbed and no longer associated with their parental infection), and *kPt* represents an actual multiplicity of phage *infection* [9,19,25,37], that is, MOI_actual_ as defined by Kasman *et al.* [38]. The phage adsorption rate constant, *k*, is the probability that a single phage within a specified volume will encounter and then adsorb a single bacterium. This value is based in part on the rate of phage diffusion along with the size of bacterial targets (Section 2.1.1). Note in this equation that phage densities are presumed to remain constant over the course of the interval, *t*, a situation that may be readily approximated particularly when bacterial densities are low [39].

The more bacteria that are present within a given environment then the more likely that a specific phage will encounter some bacterium, such that,


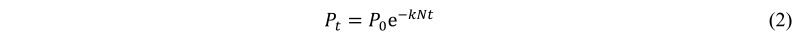


where *P*_0_ is the initial phage density and *P*_t _is that density after time, *t*. This equation in particular describes the loss of free phages as a function of bacterial adsorption. Substantial declines in phage titers will occur due to bacterial adsorption, however, only if bacterial densities are relatively high or *t* is relatively large. Consequently, and as is true also with Equation (1), for this study I employ the simplifying assumption that phage densities do not vary over time. Operationally, this means that I am placing greater emphasis on consideration of bacterial vulnerability to phages than I am on the dynamics of phage generation and loss.

### 2.2. Phage Interaction with Bacterial Arrangements

We can increase the complexity of phage-bacterial interactions by considering bacteria that are found predominantly as arrangements rather than as otherwise “free” cells (note that generally, in using the term “arrangement”, I am implying microcolony as well, that is, clonally related bacteria that by some means are found attached to one another). For example, bacteria can be arranged as doublets of cells (such as diplococci), strings of bacteria (streptococci or streptobacilli), or other, more complicated forms (staphylococci, tetrads, or sarcinae), and even, as indicated above, as microcolonies as well as biofilms. These arrangements are formed in the course of bacterial division and they differ in terms of the number of divisions that take place prior to cell separation as well as in terms of the planes of those divisions. Forming into arrangements presumably provides bacteria with selective advantages, as considered in Section 2.3.1. This is just as the specific shapes that different bacterial strains and species display, such as coccus *versus* bacillus *versus* spirillum, can be viewed as presumptively adaptive [40,41] or biofilm phenotypes can be seen as improvements in some manner upon the planktonic state [23].

Existing as arrangements, or as microcolonies, may be costly in the face of phage-mediated predation. We can consider this proposed elevation in costs as a consequence of increases in the overall target size of arrangements relative to individual bacteria, which is relevant especially in combination with increased potential for phage propagation within arrangements. At an extreme, arrangement target size could increase directly as a multiple of the number of bacteria found within an arrangement (*i.e.*, ten bacteria as a single target could be ten-times as likely to become phage adsorbed as a single bacterium). Again at an extreme, once an arrangement has become phage infected, then complete loss of all bacteria found in that arrangement could occur. In this section, I consider limitations on these extremes. I nevertheless retain the general conclusion that group living could increase bacterial vulnerability to phages.

#### 2.2.1. Increased Target Size

The likelihood of a bacterium encountering a phage, as indicated in Equation (1), is *kPt*, where *k* is a function in part of the bacterium’s target size [24,35]. If bacteria form into arrangements, then the likelihood that a specific bacterium encounters a phage may be lower due to partial shading of bacteria by other bacteria [42] or, alternatively, because of shading that results from bacterial association with surfaces. To reflect these issues, I will use the term 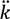 to describe reductions in phage adsorption rates to bacteria that stem from shading, such that 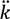 < *k*. Note that the umlaut’s intention is to imply a description of properties associated with bacterial arrangements, with the double dots literally suggestive of a diplococcus. 

The rate of phage adsorption to a bacterial arrangement can be described as *n*
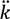
*P*, where *n* is the number of bacteria making up an individual arrangement. That is, the *target size* of an arrangement increases by a factor of *n* relative to free bacteria while at the same time decreases by a factor of 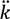
*/k*. The increase due to *n*, however, likely is greater than the decrease described by 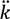/*k*, at least so long as arrangements are not sequestered within phage-excluding volumes such as (perhaps) defects in the glass walls of chemostats [29]. Larger arrangements, in other words, almost inevitably will tend to serve as larger targets for phage encounter than will either individual bacteria or smaller arrangements.

**Figure 2:**
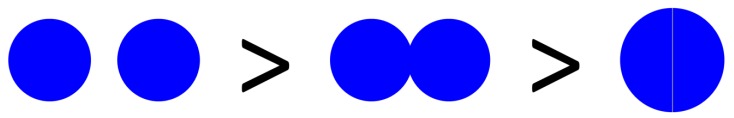
Shading of bacteria by bacteria. Shown is a progression starting with two “free” coccus-shaped bacteria (left) which is followed by a diplococcus displaying some degree of attachment (middle) that in turn is followed by a diplococcus displaying maximal attachment as well as minimized surface-to-volume ratio (right), *i.e.*, existing as a combined-volume sphere of 2^1/3^ -fold increased radius over an individual cell (see calculation, below). The left-hand lack of arrangement shows no shading whereas the right-hand arrangement shows an approximation of maximal shading for a combined spherical shape. The middle arrangement displays some intermediate degree of shading and therefore some intermediate overall target size between maximal and minimal (holding cell volumes constant). Note that the volume of a sphere, *V*_1_, is equal to 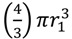. Twice its volume (*V*_2_) therefore is 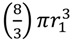, which as a sphere is equal to 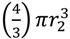. For 
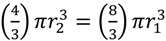
, then *r*_2 _= 2^1/3^*r*_1_. With such shading, then, diameter increases by only 2^1/3^ = 1.26 fold.

Generally it is the diameter or ‘breadth’ of bacterial targets that is crucial to determining viral contact rates [24,35]. For example, the target size of paired, spherical bacteria (diplococci) will range between ~1.26 (= 2^1/3^ = *n*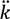), which is the increased diameter of a two-fold larger volume, and approximately two (*n*) times larger than the target size of individual cocci. These values in other words range from where shading is substantial (1.26 times) to where shading instead is minimal (~2 times; for illustration, see Figure 2). Given diversity in arrangement shape it is clear that using arrangement diameter as a proxy for target size is a simplification, though one which I retain both for the sake of mathematical convenience and because assuming that targets are spherical may be the most reasonable of default assumptions. Clearly though, and as indicated in the above calculation (Figure 2), surface area (as equivalent to the “~2 times” calculation) provides a more intuitive perspective on target size and particularly so given non-spherical as well as relatively immobile targets. The larger and more important point, however, is that in terms of target size, arrangements should be inherently more vulnerable to phage encounter than individual bacteria.

#### 2.2.2. Increased Multiplicity of Adsorption

To visualize the impact of forming into arrangements, with shading affecting phage adsorption rates, compare Equation (1) with


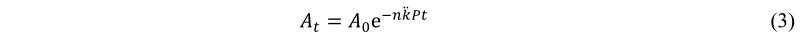


Here *A* stands for arrangement and *A_t_* is the number of arrangements that have *not* been phage adsorbed over an interval, *t*, given a constant phage density, *P*. So long as *n*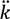 > *k* holds, then *N_t_/N*_0 _> *A_t_/A*_0_. That is, fewer arrangements will remain fractionally unadsorbed (*A_t_/A_0_*) than would individual, free bacteria (*N_t_/N_0_ )*, holding bacterial size and adsorption susceptibility otherwise constant. Here *n*
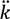
*Pt* is equivalent to MOI_actual_ for arrangements. Note though that it is my preference to instead use the term multiplicity of *adsorption*, *i.e.*, MOA, rather than multiplicity of *infection* because while an arrangement can be wholly adsorbed by a phage, subsequent infection of the whole arrangement is a more complicated process *versus* the infection of individual phage-adsorbed bacteria.

A complementary perspective on the above assertion—that is, that fewer arrangements will remain unadsorbed by phages relative to free bacteria,* N_t_/N*_0 _> *A_t_/A*_0_—is that MOA for arrangements can be up to *n*-fold higher than that for individual cells. A quantity that I will call MOA_input_ (*M*) can, after Kasman *et al.* [38], be set equal to the density of phages divided by the density of phage targets. The density of arrangements (*A*_0_), as phage targets, is expected to be *n*-fold lower than that of free bacteria, *i.e.*, *A_0_ = N_0_/n*, assuming a constancy in both cell size and total species biomass [36,43]. Holding phage numbers constant, then *M* for arrangements (*M_A_*) is expected to be *n*-fold higher than *M* for free bacteria (*M_N_*), since *M_A_ = P/*(*N_0_/n*) whereas *M_N_ = P/N_0_*. The fraction of targets expected to remain unabsorbed, in turn, is readily calculated as e*^-M^*, which is the frequency of the zero category—bacteria (*N_t_/N*_0_) or arrangements (*A_t_/A*_0_) experiencing no phage adsorption—given a Poisson distribution of phages adsorbing to targets. The larger *M* then the smaller the fraction of cells or arrangements remaining unadsorbed, and therefore *N_t_/N*_0 _> *A_t_/A*_0_ if *M_A_ > M_N_*. More precisely, we can consider instead MOA_actual_, which are *M_A_ = n*
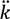
*Pt versus M_N_=kPt*. With *M* defined in this manner, then the fraction of phage targets expected to remain unadsorbed is equal to 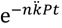 (= *A_t_/A*_0_) and e*^-kPt^* (= *N_t_/N*_0_), respectively, which are restatements of Equations (3) and (1), respectively. Note that 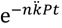 < e*^-kPt^* if as expected *n*
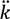
*Pt > kPt*, implying that *A_t_/A*_0_ < *N_t_/N*_0_.

These considerations come with the caveat that increases in the likelihood of arrangement adsorption that occur as a function of *n*, that is, as *n* contributes to arrangement diameter and therefore to target size, may be slowed to the extent that adsorption rates to the individual cells making up an arrangement, 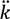, also may decline as *n* increases. It may be harder, that is, for environmental phages on average to encounter *individual* bacteria that are found within larger arrangements (such as a large microcolony) *versus* individual bacteria that are found in smaller arrangements (such as a diplococcus). Arrangement vulnerability to adsorption, given this tendency, therefore might increase *less rapidly* as a function of the number of bacteria that they contain.

#### 2.2.3. Phage Propagation within Arrangements

The expression *n*
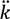
*Pt* should adequately describe the likelihood of arrangement encounter with a phage. Further, *n*
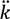
*Pt* defines the actual multiplicity, a.k.a., multiplicity of adsorption, of a bacterial arrangement with the value 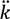 specifying inefficiencies in this phage adsorption process in comparison to free bacteria. In addition, phage adsorption to arrangements is not identical to phage adsorption to free bacteria because only a fraction of the number of bacteria making up an arrangement become initially phage adsorbed (*i.e.*, 1/*n*) rather than all of the bacteria making up a free bacterium (1/1). Furthermore, with bacterial arrangements an initial (“primary”) phage adsorption could give rise to a variety of subsequent outcomes including infection of only the adsorbed bacterium, subsequent infection of a fraction of the bacteria found within the arrangement, or indeed subsequent infection of all of the bacteria making up an arrangement. The latter result, of course, is the more costly of outcomes to the affected bacteria, just as it is in terms of prey aggregation more generally [36]. The result is that an additional assumption must be made to argue that the vulnerability of bacterial arrangements to phages can be greater than that seen for individual bacteria. This assumption, in particular, is that the efficiency of phage propagation among bacteria found within arrangements must be greater than that which can be sustained among free bacteria.

When phage densities are higher, then the likelihood of phage adsorption of a given bacterium also should be higher, as described by Equation (1). Importantly, then, the density of phages immediately surrounding a lysing bacterium, that is, as made up predominantly of those phages released from that bacterium, will be the highest phage densities that can be readily attained within a given environment. The phages released in this burst will then diffuse outward, declining in density as they do. The result should be a higher rate of phage adsorption to any susceptible bacterium found within the immediate vicinity of a lysing bacterium, but lower rates at increasing distances (assuming that phage intrinsic adsorption ability does not substantially increase over the course of environmental diffusion).

**Figure 3:**
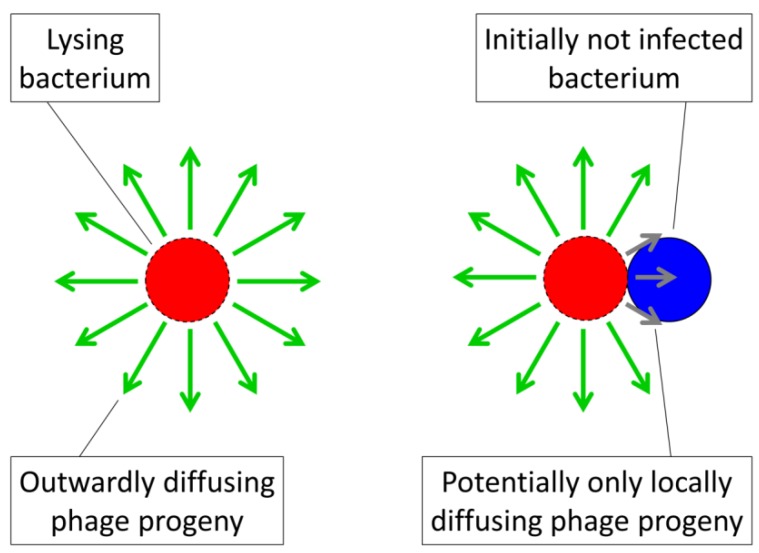
Illustration of the tendency of phages to display biases towards acquisition of locally available bacteria. Here shown to the right is phage acquisition of a bacterium (blue) that is found as part of the same arrangement as a lysing bacterium (red with dashed border). The green arrows represent outwardly diffusing phage progeny released upon bacterial lysis while the shorter, gray arrows illustrate the tendency of those phages that are released immediately adjacent to an uninfected bacterium to encounter that bacterium. Contrasting this second bacterium looming large in the vicinity of an adjacent phage burst, even at a high plankton bacterial density of 10^8^ per mL, each free bacterium (left) occupies a total environmental volume of 10^4^ µm^3^ (1 cm = 10^4^ µm, meaning that 1 mL = 1 cm^3^ = 10^12^ µm^3^, where 10^12^ µm^3^/10^8^ bacteria = 10^4^ µm^3^/bacterium). This density in turn implies an average distance between bacteria of about 10^4/3^ (*i.e.*, the cube root of 10^4^ µm^3^), or more than 10 µm, which one may compare with a typical bacterium diameter of about 1 µm. Thus, bacteria in arrangements can be not-unreasonably described as having local densities that should encourage phage adsorption with higher likelihood than that seen among planktonic, individual bacteria.

While free bacteria can randomly find themselves in the vicinity of a lysing bacterium, bacteria that are found in arrangements can be spatially constrained to that vicinity. The result is a higher likelihood of phage infection of other bacteria found within the same phage-infected arrangement than to other environmental bacteria (Figure 3). This argument is similar to an observation made by Babic *et al.* [44] that transfer of conjugative transposons among bacteria found in arrangements (chains) too can be quite efficient and this is for similar reasons, *i.e.*, a constraining of bacterial location in arrangements to within the vicinity of agents infecting the same arrangement (p. 1): “Since many bacterial species grow naturally in chains, this intrachain transfer is likely a common mechanism for accelerating the spread of conjugative elements within microbial communities.”

Analogously, greater bacterial densities found within bacterial arrangement can be viewed as possessing a higher “mass” relative to that associated with planktonic bacteria, where by “mass”—potentially confusingly—I am referring to immediately local *densities* of bacteria. This “mass” of bacteria within a bacterial arrangement would be more likely to exceed a “critical” level, such that phage propagation can be sustained, than may be achieved by an equivalent number of more locally dilute, free bacteria. This perspective is just as one can consider for nuclear fission, *i.e.*, radioactive decay, and associated chain reactions [45], which occurs more rapidly given higher “fuel” densities. Indeed, the idea of a “critical mass” can be directly equated with phage proliferation thresholds, that is, those bacterial densities at which rates of phage population growth are perfectly balanced by rates of phage loss [19,25]. Each is a description of target densities (atoms or bacteria) that can be sufficiently high that collision with targets (by neutrons or phages), in combination with subsequent proliferation (via fission or infection), balances any losses that can occur due either to interactions with nontargets or movement away from the focus “mass”. 

A target bacterium that is found in the immediate vicinity of a phage, in other words, displays a much higher local density from the perspective of that phage than may be case for bacteria that are randomly dispersed throughout an environment. A local concentration of bacterial “mass” thus can result in a high propensity for bacterial adsorption by phages that have been generated within the same “mass”. See Abedon [34], by contrast, for illustration of the relatively low propensity for phages to randomly encounter free-floating bacteria found within fluid environments; see too Hagens and Loessner [46] as well as Goodridge [47]. As default assumptions, therefore, we can view bacterial arrangements as both larger targets for phage adsorption than individual bacteria and as locally higher bacterial densities, densities that may be better able to support local phage propagation and population growth than more diffuse populations of free bacteria. Consistent with the analogy with nuclear fission, which can be controlled by the insertion of neutron-absorbing substances, we can question the efficiency of phage acquisition and then infection of seemingly adjacent bacteria, e.g., perhaps as may be separated by extracellular matrix.

#### 2.2.4. Inefficiencies in Phage Propagation

Notwithstanding proposed tendencies for phages to more readily acquire bacteria that are within their immediate vicinity, it is as noted possible for inefficiencies to exist in the sequential phage infection of bacteria co-occupying the same arrangements. To incorporate such inefficiencies into models of arrangement vulnerability to phages, I employ the term, 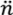. This represents the number of bacteria within an arrangement that will be lost, on average, as a consequence of phage adsorption of a single bacterium within that arrangement. This number, 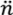, and can range up to the total number of bacteria making up an arrangement (*n*). Another way of viewing 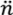, however, is that 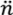 > 1 implies a phage reproductive number within a bacterial arrangement that is greater than 0 such that some degree of phage propagation within an arrangement occurs along with consequent bacterial death. If insufficient phage release from infected bacteria and/or insufficient subsequent bacterial infection occurs within an arrangement, or subsequent infections don’t happen fast enough, then complete eradication of an arrangement by an infecting phage may not happen, such that 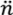 < *n*.

These ideas can be expressed as,


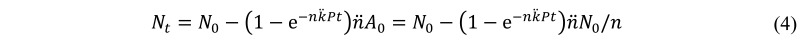


**Figure 4:**
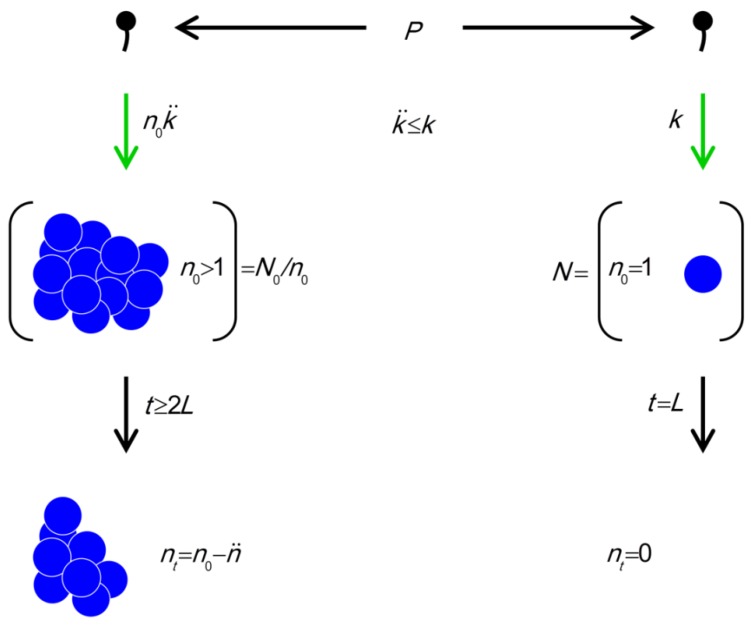
The model. Parameters include *P* (density of phages in environment), *k* (phage adsorption constant), 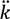 (phage adsorption constant considering reductions due to shading of bacteria by bacteria found within bacterial arrangements), *n* (number of bacteria found per arrangement), *N* (bacterial density of overall environment), *L* (phage latent period, which is the duration of a phage infection), and 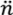 (number of bacteria per arrangement lost subsequent to phage infection of one cell in the arrangement). Likelihood of phage adsorption of bacterial arrangements is *n*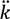 and density of arrangements within environments is equal to *N/n = N_0_/n* (or indeed *n*_0_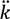 and *N_0_/n_0_*, respectively, to reflect that *n* changes as a function of time in the figure). The inequality *t ≥* 2*L* indicates how phage acquisition of bacteria within a bacterial arrangement, according to this model, involves at least two sequential rounds of phage infection. The absence of cells in the lower right is intentional as too is the reduction in cell number to *n_t_* in the lower left. Both of these reductions in cell number, going from middle to bottom, indicate phage-induced bacterial lysis.

Where (1 - 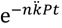) is the fraction of arrangements that become phage adsorbed over some interval, *t*, and 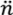 is the number of bacteria per arrangement that are lost to this adsorption (assuming, for simplicity, that 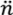 is independent of the actual multiplicity of phage adsorption to a given arrangement). Were 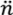 = 1, then though arrangements are more likely to be adsorbed than free cells, nevertheless no more bacteria would be lost per arrangement adsorption. Indeed, to the extent that the initial bacterial infection is less likely, that is, given 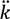 < *k* along with 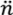 = 1, then overall existence as an arrangement could result in *less* vulnerability to phages rather than more. Such a situation would occur, for example, were phage infections abortive or perhaps could result instead were infections substantially reduced in burst size or extended in latent period such that phage propagation through an arrangement were substantially impaired. Alternatively, the equality 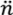 = *n* would imply complete arrangement loss following each phage adsorption of an arrangement. The parameter, 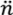, is thus a description of arrangement vulnerability to phages post-adsorption, ranging from minimal (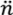 = 1, or even 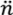 = 0) to maximal (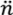 = n ). A visual summary of the models represented by Equations (1) and (4) is presented in Figure 4.

#### 2.2.5. An Important Special Case

Equation (4) is less applicable given higher levels of phage adsorption, as can occur over longer periods. This is because, as noted, Equation (4) fails to take into account the impact, on the overall fraction of bacteria that are lost, of multiple phage adsorptions of individual arrangements. That is, for instance, we might have an expectation of greater bacterial loss with greater levels of arrangement adsorption by environmental phages under conditions where otherwise 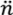 < *n*, but Equation (4) does not reflect that if one adsorbing phage fails to clear an arrangement then perhaps more than one adsorbing phage will, with greater likelihood, succeed in doing so. One way to address this concern is simply by setting 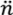 = *n*, that is, an assumption of complete arrangement loss per primary phage adsorption.

Alternatively, one can limit one’s considerations to circumstances in which phage multiplicities of adsorption to arrangements are relatively low (<<1). In such cases the equation can be simplified as


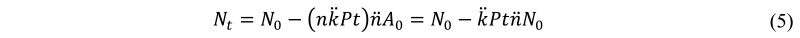


which compares, for a free bacterium, with,


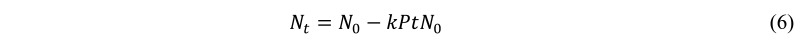


Note that *N_t_* (as defined by Equation (5)) is greater than *N_t_* (as defined by Equation (6)) when 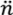 > *k*/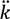. That is, when this inequality holds then bacteria found within bacterial arrangements are more vulnerable to phages than are phage-susceptible bacteria that are “free”. In words: Bacteria found within arrangements are more susceptible to phage attack if losses due to existence within a phage‑adsorbed arrangement, 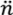, are greater than increases in *individual* bacterial vulnerability to “primary” adsorptions that come from *not* existing within an arrangement (that is, *k/*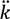 where *k* > 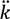).

If a bacterium is half as likely to be subject to primary phage adsorption when found within an arrangement (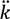/*k* = 0.5 such that *k*/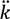 = 2) but each primary adsorption results in the loss of ten bacteria (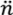 = 10), then the resulting 10 > 2 would imply that arrangements are more vulnerable to phages than are free bacteria. Indeed, in this example bacteria found within arrangements would be five times more vulnerable. Note further that if arrangements can reduce 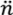 to 1, then arrangements will be expected to display lower vulnerability to phages than free bacteria so long as individual bacteria found within arrangements are less vulnerable to primary phage adsorptions than free bacteria (*i.e.*, again, such that *k >* 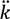). This is an observation that could very well explain the utility of abortive infection systems to bacteria, *i.e.*, phage-resistance mechanisms in which both adsorbed bacteria and adsorbing phages die [30]. Alternatively, for 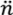 = 0, which is the case given successful bacterial display of, for example, antibacterial restriction-modification systems, then biologically the inequality no longer holds, *i.e.*, 0 > *k/*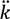. Logically, though, in this case arrangements should be no more or less vulnerable to phages than free bacteria since, in fact, neither would be vulnerable. See Table 1, under “Higher” phage densities for summary. Note, though, that calculations relevant to the “Lower” phage densities portion of the Table, *i.e.*, Equation (7), are not discussed until Sections 2.3.3 and 2.3.4.

**Table 1:** Summary of predictions as a function of phage densities in environments and phage potential to acquire bacteria sequentially within bacterial arrangements (Recall in interpreting the table that the inequality,* N_t _/ N*_0 _> *A_t _/ A*_0_, implies greater success over time in the face of phage-mediated predation for free bacteria *versus* bacteria found within arrangements while *N_t _/ N*_0 _< *A_t _/ A*_0_ implies the opposite). Generally, *k/*
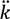
≥ 1. Calculations relevant to lower phage densities, as found in the bottom portion of the table, are not discussed until Section 2.3.3 and especially section 2.3.4.

Environmental Phage Density (*P*)	Phage Propagation Ability Through Arrangements ( 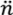 )			
	Higher	Lower		
**Higher** (bacterial losses dominate dynamics)	For 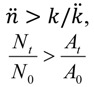 [lesser or no impediments to phage propagation within arrangements]	For 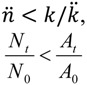 [impediments less than absolute]	For 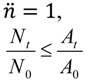 [e.g., abortive infections]	For 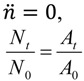 [e.g., phage restriction]
**Lower** (bacterial gains dominate dynamics)	For 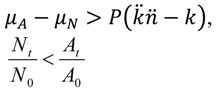 [which, as *P →* 0, is more likely]	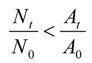	[assuming phage-independent advantages to arrangement formation, *i.e.*, *μ_A_* - *μ_N_ >* 0, and that *μ_A_* - *μ_N_ > P*( 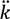 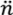 -*k*)holds, which is likely given both *P →* 0 and 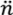 *→* 0]	

### 2.3. Utility of Group Living in Light of Phages

Above I argue that the likelihood of adsorption by any given phage can be lower for individual bacteria found within arrangements (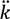) than for free bacteria (*k*) but nonetheless that arrangements can be more vulnerable to exploitation by phages. If we assume that arrangements nevertheless provide bacteria with selective benefits, then it should be possible to consider how large these benefits must be to offset costs stemming from this presumed increased susceptibility of arrangements to phage infection. Before moving on to that issue, however, I first address two underlying considerations, (1) how existence within clonal associations in fact might benefit bacteria and (2) experimental evidence that phages can propagate through bacterial arrangements and/or microcolonies.

#### 2.3.1. Selective Benefits of Living in Arrangements

Fitness advantages accrued by bacteria from living within groups generally can result from increased short-term growth rates, greater long-term rates of reproduction, increased population resistance to extinction, or greater competitive ability in terms of more effectively sustaining population densities. The latter might be accomplished by gaining better access to nutrients. Adherence to surfaces in conjunction with subsequent growth as a microcolony, for instance, can retain a bacterial population within the vicinity of key resources, such as flowing water [48].

Continued association of cells following division similarly may allow for more effective penetration into resource-supplying substrates, *i.e.*, burrowing, particularly as seen among filamentous microorganisms growing in spatially structured environments such as soils. Aggregated cells also will tend to have larger collective activity domains [49]. This may be beneficial to bacteria by allowing better retention of extracellular regulatory molecules, particularly such as towards quorum sensing [1,50], or for the concentrating of other beneficial extracellular factors such as exoenzymes or exotoxins. Reduced levels of sharing with unrelated organisms of any molecules generated by these extracellular factors may be possible given bacteria association within arrangements and this may be so simply given greater densities of those cells within a given microvolume.

Growing in a single location can allow for more effective interspecific interactions [49] including crossfeeding [51] as well as development of closely spatially integrated microbial consortia [52,53]. Such associations might be enhanced by cells that grow to higher densities within a specific location, including as cell arrangements or instead as cells that simply fail to disperse and thereby form into microcolonies (or, instead, simply don’t disperse very far). More generally, the development of favorable physiochemical gradients might be more readily achieved when cells are living within groups consisting at least in part of related individuals, *i.e.*, as consistent with the above-noted idea of cellular aggregates possessing larger collective activity domains *versus* bacteria that are not found in association with related bacteria. The result may be a higher potential to contribute to synergistic interspecific interactions. Indeed, “Any transfer between two such organisms will be immensely inefficient unless the concentrations of cells and substrates are high… Such interactions can take place between groups of bacteria where the size of the group means an enlarged activity domain and the retention of reactants in the vicinity of the group.” [49] (pp. 56 and 58).

Bacteria can display movement when found within disfavorable environments, such as can be associated with planktonic or actively dispersing cells, and then cease such movement, such as through adherence to a surface, when found within a favorable environment. This is a behavior that, minimally, is equivalent to the concept of orthokinesis, that is, where the speed of an organism is modified by external cues or, indeed, thigmokinesis, where speed is modified by degree of physical contact with something else. Included among favorable environments and external cues can be other microbial species from which a given bacterium can derive benefits. Association with other microorganisms on a surface, rather than being stable, can as a consequence of thigmokinesis instead be associated with the invasion of later-arriving organisms. The result can be an interspecific competition for space or resources that can put a premium on competitive ability, which in turn may benefit from more effective arrangement or microcolony formation [50,54,55]. Full or partial replacement of one microbial species with another on surfaces more generally is an example of ecological succession, as has been documented particularly well in terms of dental biofilms [49,56].

Cellular clones growing in a single location have the potential to interact with each other cooperatively towards mutual benefit and do so in ways that are less available to free bacteria. This can include elevation of colonies into more nutrient-rich microenvironments [48], certain degrees of cellular differentiation and/or physiological specialization, fruiting body formation, and even seemingly intentional cell cannibalism under starvation conditions [1,57]. Perhaps consistent with this idea of bacteria being able to mutually cooperate when living within single-species groups, cells growing as biofilms are known to display an increased resistance to toxic chemicals found in their environments, which clinically can include both antibiotics and disinfectants [3,4]. Increased resistance may also be seen given association other microbial species [58]. Extracellular polymer production itself is often described as a potentially cooperative activity among related, physically associated bacteria [55]. 

Growth as arrangements may give rise to a greater resistance to engulfment by protozoa, and biofilms otherwise may be more resistant to protozoa-mediated predation than planktonic bacteria [59]. The resulting adaptations, such as “resisting ingestion, by becoming too large or too long… making themselves inaccessible, by growing in aggregates or biofilms”, [41] (p. 3), however, may conflict with avoidance of phages by these same bacteria. Biofilm formation can also serve as a means of immune system resistance during infections by bacterial pathogens and this is due at least in part to interference with the action of phagocytes, though not necessarily solely via a direct blocking of bacterial engulfment [60,61,62]. More generally, utility that comes with being larger, whether individually per cell or instead as a consequence of cell-to-cell associations, is exploited by most non-bacterial cellular organisms including animals, plants, fungi, and protists.

#### 2.3.2. Susceptibility of Bacterial Arrangements and Microcolonies to Phage Exploitation

To what degree are bacteria living in arrangements in fact phage susceptible? This question must be addressed particularly since assertions have been made that biofilm formation serves bacteria as an inherently phage-resistant state, as I review elsewhere [9]. Costerton *et al.* [63] in particular noted that (p. 440), “the gellike state of the predominantly polysaccharide biofilm matrix limits the access of antibacterial agents, such as… bacteriophage… Therefore, biofilm bacteria are substantially protected from… bacteriophage…” Elsewhere [8,9], however, I review the substantial potential, under experimental conditions, for phages to in fact considerably impact biofilms. Phages may not be well equipped to drive biofilm bacteria completely to extinction in the course of propagating on those bacteria, and phages that specialize in targeting biofilm bacteria may not always be highly prevalent in environments. Nevertheless, there is no evidence that I know of that would appear to indicate that biofilms are *not* relatively susceptible, or in many cases even highly susceptible to exploitation by phages.

As with the phage potential to clear bacterial biofilms, evidence also exists that phages can propagate through bacterial arrangements. Barron* et al.* [64], for example, state that “the phage released from a single coccus may infect other cocci in that chain.” The evidence supporting that claim is an observation by Friend and Slade [65]. They found that one-step growth curves on a group A streptococcal strain in fact were two-step in practice, implying an initial arrangement adsorption followed by post-burst adsorption within the same arrangement; see also earlier work by Kjem, 1958 and 1964, as cited by Friend and Slade, as well as work by Fischetti* et al.* [66]. In the Friend and Slade study, two-step curves were then reduced to one-step curves through the separation of streptococci into individual, that is, “free” bacteria using sonication.

The tendency for phage plaques to be clear, particularly in their centers, also can be viewed as an indication of the tendency for phages to prevent phage-sensitive bacteria from propagating in the immediate vicinity of phage bursts. Indeed, plaque formation explicitly occurs within a context of phage penetration into and subsequent clearance of the bacterial microcolonies that make up bacterial lawns [67]. Consistently, Doolittle *et al.* [68] observed phage propagation within single-species bacterial biofilms, describing the dynamics as plaque-like. Conversely, plaque cloudiness can signify limitations on the ability of phages to clear bacterial microcolonies [9,13,69]. It should *not* be controversial therefore that phages can propagate to at least some extent into bacterial arrangements or microcolonies. Indeed, in phage therapy it is often assumed that phages can be quite adept at propagating through bacterial biofilms, a process that I have described elsewhere as an ‘active penetration’ [19]. These claims all come with the caveat, however, that such active penetration is not necessarily going to be the case for every combination of phage, bacterial arrangement, and circumstance.

#### 2.3.3. Phage-Mediated Costs of Existing as Arrangements

For the sake of mathematical convenience, I consider especially the phage impact on instantaneous bacterial population growth rates rather than other aspects of bacterial fitness in addressing the extent to which phages might affect bacterial arrangements. Focusing on instantaneous growth rates, in particular, greatly simplifies the mathematics while at the same time avoids consideration of the difficult issue of exactly how bacterial arrangements propagate over longer time periods. The conclusions I reach, however, should be qualitatively applicable to other situations.

These considerations of phage impact on the instantaneous population growth of bacteria existing within arrangements can be easily formulated as a differential equation in which changes in bacterial density (*N*) are considered in terms of rates of cell division (*μ*) *versus* declines due to phage adsorption,


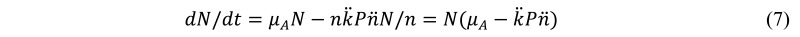


which compares with


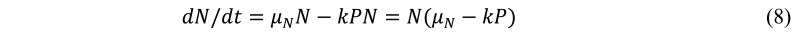


for free bacteria. Note that *μ_A_* and *μ_N_* are growth rates of bacteria associated with arrangements and free bacteria, respectively.

The expression *n*
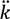
*P*
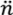
*N/n* in Equation (7), or simply *kPN* in Equation (8), describes those bacteria that have been lost from the unadsorbed bacterial pool, *N*, as a consequence of phage adsorption either of themselves (Equation (7) and Equation (8)) or of the arrangement in which those bacteria are located (Equation (7)). As noted above (*i.e.*, *A*_0_ = *N*_0_/*n* in Section 2.2.2), the expression *N / n* as found in Equation (7) is a description of the density of arrangements consisting of *n* bacteria that are found in the environment in question. Also as above, the parameter 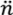 describes the number of bacteria that will be lost to phage infection given phage adsorption of an arrangement. Lastly, *n*
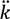
*P* is a description of the per-arrangement rate of bacterial adsorption by phages given an environmental phage density of *P*.

Per bacterium, Equation (7) thus describes adsorptions that occur at a rate of 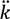
*P* and each of those adsorptions results in a loss of 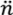 bacteria. This compares with the rate of loss of individual bacteria as described by *kP* in Equation (8). The expression, *μN* with either subscript, by contrast, describes in both equations the gains in bacterial density that occur as a consequence of bacterial replication. Thus, If 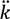
*P*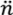 > *µ_A _*, then the bacterial population will experience a net decline in number whereas 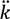
*P*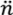 < *µ_A_* indicates net gains and 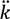
*P*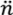 = *µ_A_* defines a steady state. For Equation (8) the equivalent expressions instead are respectively *kP > μ_N _*, *kP < μ_N _*, and *kP = μ_N_*, where *P* in the latter can be described as an inundation threshold or even phage minimum inhibitory concentration, that is, of bacteria [25]. In the absence of phages, the bacterial population will simply grow at rates specified by *μ_A_* or *μ_N_*.

In Equation (7) the dynamics of microcolony formation are not considered and nor are various other complications such as multiple adsorption by “environmental” phages of individual bacterial arrangements, where by “environmental” I am distinguishing those phages defining *P* (=environmental) from phages that instead are explicitly propagating through bacterial arrangements. What Equation (7) nevertheless indicates is that the phage impact on bacterial arrangements varies as a function of phage density (*P*) in combination with the susceptibility of individual bacteria making up an arrangement to phages (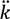

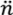
). Specifically, the larger 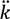
*P*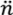 then the more bacteria found within arrangements that are lost to phage infection. For example, twice as many bacteria will be lost per unit time for bacterial arrangements (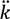
*P*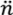 from Equation (7)) *versus* free bacteria (*kP* from Equation (8)) if 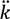
*P*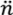 /*k* = 2. 

Alternatively, a total of 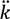
*P*
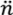
*/k*-fold more arrangement-associated bacteria will be lost *versus* free bacteria for any given phage density, *P*. Forming into an arrangement, and thereby incurring costs of additional vulnerability to phages, therefore should be worthwhile to bacteria *only* to the extent that *μ_A_*, or some other measure of bacterial fitness, increases as a consequence of group living to a larger extent than bacterial fitness decreases as a result of incurring a greater spatial vulnerability to phages, *i.e.*, as described by 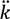
*P*
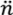
* /k*.

#### 2.3.4. Importance of Reduced Vulnerability to Phages

The extreme situations with regard to Equation (7) are as follows: (i) If no phages are present in an environment (*P* = 0) then there will be no phage-associated cost to arrangement, microcolony, or biofilm formation (in which case no advantage is required from group living to offset costs of phage adsorption) or (ii) if phages are present at effectively infinite densities (*P = ∞*), or simply sufficiently high densities, then potentially no amount of phage-independent benefit to group living could offset increases in phage-associated vulnerabilities. The latter situation is just as dire for free bacteria, however, *i.e.*, for which 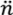 = 1 but nonetheless where it is possible for 
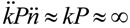
.tif (Equation (7) *versus* Equation (8)). At a minimum, however, *μ_A_* = 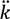
*P*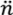 is necessary for bacterial fitness, as measured here in terms of increases in bacterial growth rates, to offset costs due to phage adsorption, and this compares with *μ_N_ = kP* for free bacteria; see Abedon and Thomas-Abedon [19] along with references cited, or Abedon [25], for derivation of the latter. 

For circumstances in which 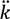
*P*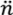 > *kP*, then the growth rate or other measure of the fitness of bacterial arrangements must be greater than that of free bacteria by that amount, e.g., *μ_A_*-*μ_N_* > 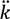
*P*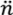 - *kP =P*(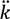

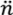
 - *k*), to offset increased phage-associated costs that are borne by bacterial arrangements. This fitness improvement, however, need not be substantial unless phage densities (*P*) are also substantial. Thus, as 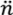 or 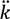 increase so too does the potential for phages to block the evolution of bacterial arrangements, but at the same time such increases do not serve as absolute blocks on this evolution. The alternative perspective is that given sufficiently high phage densities—but not too high, as indicated in the previous paragraph—then evolution could tend to favor reductions in 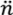 even if bacteria otherwise experience benefits from forming into arrangements, that is, reduced formation of arrangements could serve as a bacterial anti-phage strategy. In simple terms, a coccus might encounter a phage approximately half as often as a diplococcus. 

A generalization on these considerations is that bacteria that have formed into arrangements will have to avoid, on a per-bacterium basis, equivalent increases in vulnerability to phages in order to partake of whatever net advantages may be associated with forming into arrangements. This reduced vulnerability, furthermore, can result either from existing in environments in which phage densities are low (where phage-independent bacterial fitness “gains” can dominate competitive dynamics) or, alternatively, from greater resistance by individual bacteria to phage attack when phage densities are high, that is, when phage-mediated losses dominate competitive dynamics [30,31]. This idea of predator-independent aspects to fitness dominating prey evolution when predator densities are low (here bacteria and phages, respectively) but predator-resistance dominating when prey densities are high is a standard conclusion from community ecology. It is also seen in phage-bacterial chemostat studies as phage-sensitive bacteria, if they possess a growth-rate advantage to phage-resistant bacteria, out-compete phage-resistant bacteria when phage densities are lower but those same phage-sensitive bacteria are at a competitive disadvantage if phages instead are more numerous [70,71]. See Table 1 for a general summary of fitness expectations for arrangements *versus* free bacteria given higher *versus* lower arrangement vulnerability to phages.

#### 2.3.5. Reduced Bacterial Densities as Phage-Resistance Strategy

Perhaps the simplest approach to reducing arrangement vulnerability to phages would be for those bacteria to exist at lower population densities, population densities, that is, which are insufficient to support phage population growth to levels at which those phages can substantially impact arrangement fitness. In other words, bacteria that are *less* able to attain “winner” densities within a given environment—as in “kill the winner” (Section 1)—may as a consequence be better able to exploit biofilm niches. This idea is similar to the conclusions of Ioannou *et al.* [36], as described in Section 2.1.1, who suggest that one means by which prey species can offset the costs of possessing greater individual sizes is by displaying lower population densities such that, as a prey type, they effectively are a greater distance, on average, from their predator and therefore less likely to be desirable to the predator. A major difference between the scenario presented by Ioannou *et al.* and that of kill the winner, however, is seen in terms of the degree of specialization of the predator species. For phages, specialization can be extreme such that bacteria phage-susceptibility types can “hide” by failing to support phage replication to inundative densities, that is, by not being “winners”. For the visual predators considered by Ioannou *et al.*, by contrast, it is not that prey avoid predation by impacting predator densities but instead that they avoid being consumed by being less visible to predators that already exist at some more or less fixed density.

Biofilms often can occupy only a small fraction of total environmental volumes, such as in aquatic environments. In such circumstances, biofilm bacteria as a consequence may be inherently unable to achieve winner-level densities. This low potential, furthermore, may be particularly the case to the extent that biofilms consist of mixed populations of bacteria rather than monocultures of specific phage susceptibility types, thereby implying even lower densities of individual bacterial phage susceptibility types than available surfaces might maximally hold. Alternatively, biofilms found within highly structured environments, such as undisturbed soils [18], may over time tend to be mostly sequestered within micro-localities away from phages to which they are susceptible. Physically associated clonal groups of bacteria, such as can make up biofilms, in other words, may not be so much inherently resistant to phage attack as either relatively unexposed to specific phages or inherently less able to support the population growth of those phages to inundative densities [9].

An additional possibility may hold and that is that it is particularly those bacteria which, as a matter of luck, are resistant to all phages present within environments that may attain “winner” densities, either free or in association with arrangements. This latter idea is ecologically similar to the domination of animal-rich ecosystems by plants that are resistant to forage by those herbivores which happen to be present within an environment [72]. That is, bottom-up control on bacterial density (nutrient availability) rather than top-down control (predator prevalence) would be expected to operate within ecosystems where predators are lacking whereas a combination of bottom-up and top-down control may hold when differences exist among prey in terms of their resistance to predation [70]. We might therefore predict a surfeit of bacteria existing as arrangements under three distinguishable circumstances: (i) where the abundance of specific phage susceptibility types of bacteria generally is low (potentially top-down control, ‘kill the winner’, and high bacterial diversity); (ii) where phage‑resistant organisms dominate (combination of bottom-up and top-down control, and potentially lower bacterial diversity); or (iii) where phages simply are absent (bottom-up control with the diversity of bacteria therefore determined by factors other than phage-mediated predation).

## 3. Experimental Section

See Results and Discussion.

## 4. Conclusions

Group living bestows benefits—else why live in groups?—but also engenders costs. One cost comes from an increased vulnerability to exploitation that group living creates, such as a greater potential for infection of individual bacteria by bacteriophages. Here I have provided a simple model for quantifying those costs, one that points to the idea that bacterial arrangements along with microcolonies and biofilms may persist particularly under circumstances where high rates of lytic phage infection is unlikely. This conclusion is broadly consistent with Murray and Jackson’s [24] suggestion, pp. 113 and 114, that “…for any given viral concentration, a large particle is more likely to have a virus reach its surface… Any given virus, however, is far more likely to reach a small, presumably bacterial, particle than a larger one in the ocean because of the greater abundance of small particles…” That is, individual prey size and vulnerability are not the only variables controlling the susceptibility of prey *populations* to predators, with prey population density also playing a key ecological role.

This perspective contrasts with notions that group living among bacteria might *directly* serve as a means of avoiding phage-mediated predation. Observation of the existence of specific bacterial types at high environmental densities and particularly as arrangements, microcolonies, or biofilms, however, would suggest the existence of effective mechanisms by which their vulnerability to phages has been reduced, just as pathogen-resistance mechanisms have, of course, evolved in multicellular organisms such as animals [32]. One reasonable scenario explaining such a situation is that diversity may exist within bacterial communities in terms of phage susceptibility even when that diversity is not superficially obvious. In this case, densities of individual bacteria types wouldn’t be as high as they would appear—perhaps as a consequence of “kill the winner” (loss of bacterial population existing at higher environmental densities)—because densities of specific phage susceptibility types would not be appreciably high. That situation represents the default assumption that one might make with regard to large, relatively open systems, that is, stabilizing frequency-dependent selection acting on rare phage susceptibility types that results in substantial bacterial diversity particularly in terms of phage resistance [73].

Alternatively, phages to which high-density bacterial populations are sensitive simply may not have reached those bacterial populations. That situation represents the default assumption that one might make with regard to small, relatively closed systems. The utility of phage therapy as an antibacterial strategy is that in many instances the infection to be treated can be described in the latter terms: small, relatively closed systems, such as localized or even systemic bacterial infections. Efficient bacterial eradication, including of bacterial biofilms, often can be achieved, therefore, simply by “opening” these systems sufficiently to phages, and the phage therapy literature, such as in terms of successful clinical treatment of infections in humans [74,75], appears to be consistent with that scenario.
